# Supplementary material for: MetaRibo-Seq measures translation in microbiomes
Source: Nat Commun. 2020 Jun 29;11:3268. doi: 10.1038/s41467-020-17081-z (PMC7324362; doi:10.1038/s41467-020-17081-z)
Supplement: Supplementary file 10 — Supplementary Data 7 [file 41467_2020_17081_MOESM10_ESM.zip › File2/Confidence_High_Taxonomy/87404_out.krona.html]

Javascript must be enabled to view this page.

members
magnitude
magnitudeUnassigned
count
unassigned
taxon
rank

87404\_out

2

2
superkingdom
2

976
phylum
2

2
class
200643

171549
2
order

1
family
171552

genus
1

SRS145503\_contig\_number\_contig-100\_4778.32782
838

815
family
1

genus
1
816

310297

SRS015663\_contig\_number\_contig-100\_311.45891
1
species
